# Localisation abdominale d’un dermatofibrosarcome de Darrier et Ferrand: à propos d’un cas

**DOI:** 10.11604/pamj.2021.38.365.26770

**Published:** 2021-04-14

**Authors:** Issam Loukil, Amine Zouari, Bassem Abid

**Affiliations:** 1 Service de Chirurgie Générale, Tataouine, Tunisie,; 2 Service de Chirurgie Générale, Sfax, Tunisie

**Keywords:** Dermatofibrosarcome, clinique, chirurgie, pronostic, à propos d’un cas, Dermatofibrosarcoma, clinical, surgery, prognosis, case report

## Abstract

Le dermatofibrosarcome est une tumeur cutanée rare d´aspect morphologique trompeur et inconnu pour la plupart des médecins. Le retard diagnostique peut conditionner la prise en charge et affecter le pronostic. Nous rapportons le cas d´un jeune patient qui a présenté des lésions protubérantes de la paroi abdominale pris à tort pour des kystes bénins. L´exploration radiologique a révélé une masse tissulaire du plan graisseux sous cutané suspecté d´être un fibrosarcome. Cette masse a été réséquée avec une marge de sécurité macroscopique. L´étude anatomopathologique a confirmé le diagnostic de dermatofibrosarcome. Le contrôle clinique et radiologique à distance n´a pas objectivé de récidive. Ce cas nous permet d´éviter de passer à côté d´une tumeur rare qui nécessite une prise en charge spéciale.

## Abstract (en)

Dermatofibrosarcoma is a rare skin tumor. Morphological characteristics can be misleading and unknown to most of physicians. Diagnostic delay may affect patient´s management and prognosis. We here report the case of a young patient with lesions protruding from the abdominal wall mistaken for benign cysts. X-ray examination revealed subcutaneous adipose tissue mass. This was suspected of being a fibrosarcoma. The mass was resected with a macroscopic safety margin. The anatomo-pathological study confirmed the diagnosis of dermatofibrosarcoma. Clinical and radiological follow-up examinations didn´t show any recurrence. This study highlights the importance of suspecting this rare tumor requiring specialist treatment.

## Introduction

Le dermatofibrosarcome (DFS) est une tumeur rare qui représente 0,1 à 0,18% des tumeurs cutanées malignes. L´incidence estimée est de l´ordre de 0,8 à 4,2 cas / millions d´habitants /an. Son aspect est trompeur, évoquant une cicatrice chéloïde et sa méconnaissance par la plupart des médecins sont responsable souvent d´un retard de diagnostic. Sa gravite est liée à son agressivité locale, son caractère récidivant et sa transformation sarcomateuse maligne exceptionnelle [1,2].

## Patient et observation

Nous rapportons le cas d´un patient âgé de 40 ans, sans antécédents pathologiques notables, qui consulte pour une masse abdominale évoluant depuis quelques mois. A l´examen, on trouve une masse oblongue protubérante de 6cm de grand axe para-ombilicale gauche, qui est non douloureuse et mobile par rapport au plan profond. Le reste de l´examen est sans particularités notamment pas de signes digestifs associés. Le Tableau 1 résume la chronologie de la maladie.

**Tableau 1:** chronologie de la maladie et des examens complémentaires

15/10/2019	25/10/2020	05/11/2020	15/11/2020
Consultation pour une masse protubérante de la paroi abdominale évoluant depuis quelque mois	Imagerie: échographie abdominale IRM Abdominale	Endoscopie: fibroscopie œso-gastro-duodénale Coloscopie totale	Opération

**Figure 1:**
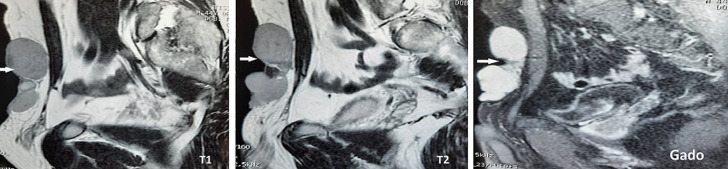
coupe sagittale, hypointense en T1 et hyperintense en T2, rehaussement de la masse sans signes d´extension aux organes intra-abdominaux en sagittale T1 avec saturation de la graisse et injection de gadolinium

Une première exploration morphologique par échographie a montré une masse mal limitée de 6cm de grand axe, d´écho-structure hétérogène infiltrant toutes les tuniques de la paroi abdominale. Pour mieux spécifié cette tumeur et pour étudier ces rapports, un complément d´exploration par imagerie par résonance magnétique (IRM) abdominale a été demandé. Cet IRM montre une masse tissulaire multiloculaire de la paroi abdominale antérieure péri-ombilicale, bien limitée, mesurant 7cm de hauteur, 6cm de largeur et 2,5cm d´épaisseur. Elle est strictement limitée au plan graisseux sous cutané de signal homogène, hyperintense en T2 et hypointense en T1, se rehaussant après injection de Gadolium sans signes d´extension aux organes intra-abdominaux (Figure 1). Devant ce tableau, une tumeur fibromateuse type desmoïde a été évoquée et une colonoscopie a été demandée à la recherche de polypes coliques associés, revenue sans anomalie.

**Figure 2:**
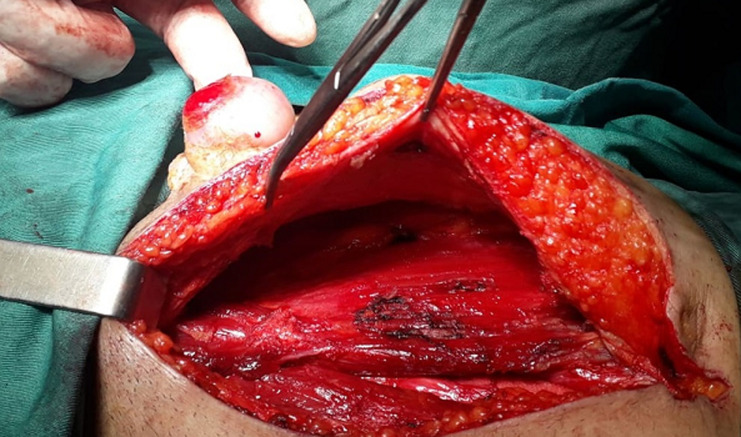
ouverture médiane et déconnexion de la masse de la cavité abdominale

**Figure 3:**
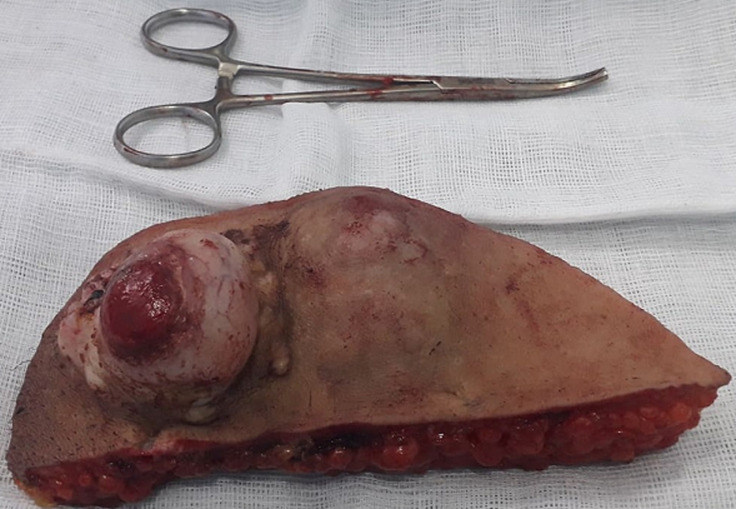
pièce de résection en monobloc

Le patient a été opéré par incision médiane sous ombilicale emportant en monobloc la masse tumorale comportant le plan cutané, sous cutané, l´aponévrose superficielle et quelques fuseaux du muscle droit de l´abdomen avec une marge chirurgicale macroscopique de sécurité de 1cm en latéral et en profondeur (Figure 2, Figure 3). L´exploration peropératoire de la cavité abdominale n´a pas montré de localisations secondaires et la fermeture pariétale n´a pas nécessité de réfection prothétique ni de geste de reconstruction.

**Figure 4:**
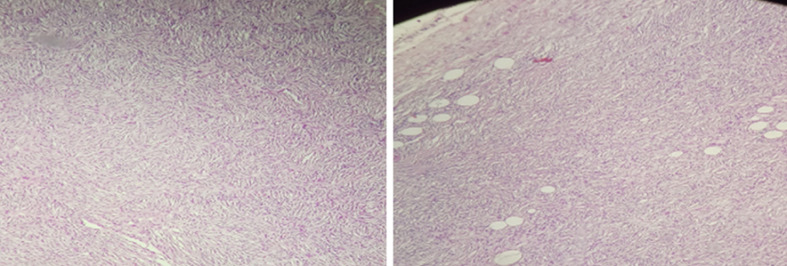
prolifération mésenchymateuse en fuseau cellulaire d´architecture storiforme (en rayon de roue), dissociation des lobules adipeux en profondeur (pathognomonique)

**Figure 5:**
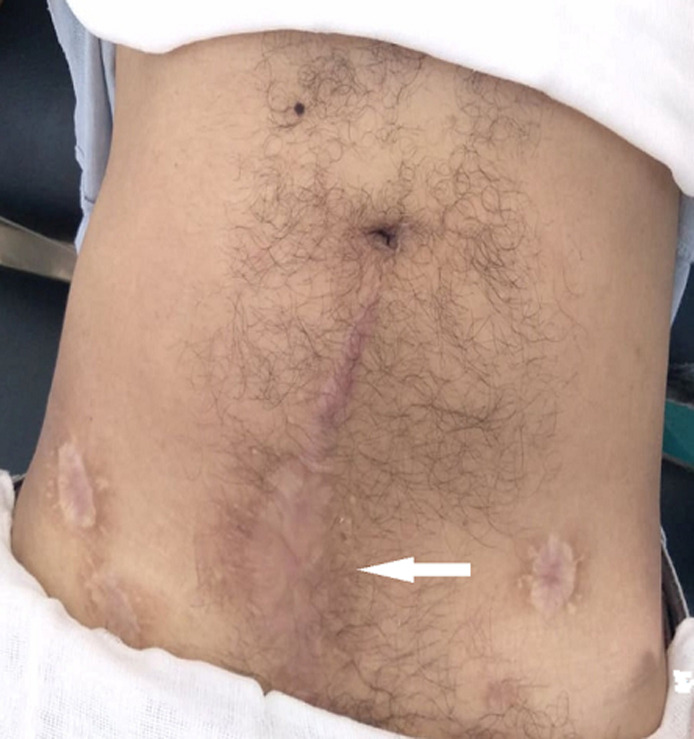
image prise à neuf mois en postopératoire

L´étude histologique de la pièce opératoire a montré une prolifération mésenchymateuse en fuseau cellulaire d´architecture storiforme (en rayon de roue) (Figure 4) et une dissociation des lobules adipeux en profondeur évocateur de DFS, avec marge de résection microscopique saine. Le dossier a été présenté en réunion de concertation pluridisciplinaire et un traitement adjuvant postopératoire n´a pas été indiqué. Un contrôle clinique et échographique à 9 mois n´ont pas montré de signes de récidive locale (Figure 5).

## Discussion

Le point fort de cette publication est qu´il rapporte un cas rare de tumeur cutanée d´aspect trompeur. Les cas rapportés à travers la littérature ne sont pas nombreux et la prise en charge ne suit pas un consensus. C´est une tumeur mésenchymateuse cutanée de bas grade à développement intradermique, qui touche surtout l´adulte jeune de 20 à 50 ans, avec une légère prédominance masculine rapportée par certains auteurs. Elle touche aussi bien les sujets de race blanche que ceux de race noire [1,3]. L´étiopathogénie reste un mystère. Elle survient après un traumatisme local dans 10 à 20% des cas qui pourrait aggraver un désordre préexistant et peut survenir aussi sur une peau saine sans dermatose préexistante ni traumatisme local comme pour notre cas [2-4]. Certains auteurs ont évoqué le thème de l´hérédité, et ont rapporté des cas chez les enfants et des cas congénitaux qui seraient en rapport avec le gène de fusion COL 1A1 - PDGFß lié à la translocation des chromosomes 17 et 22 (t (17; 22) (q22; q13)) [1,5,6].

Le DFS peut toucher tout le corps humain. Le siège tronculaire est le plus fréquent de 50 à 60% des cas. La taille varie selon les publications entre 1 à 5cm, pouvant atteindre 30cm dans la série de Hammas [3,4]. Cette lésion est douloureuse dans 10 à 25% des cas, par l´effet de masse quand elle augmente de volume et par la présence d´ulcérations hémorragiques. La plupart des auteurs rapporte l´évolution clinique en deux stades. Au départ, au stade infiltratif, la lésion se présente comme une plaque indurée, recouverte d´une peau normale, de couleurs rosée ou violacée, bien limitée et mobile par rapport aux plans profonds. Plus rarement, elle se présente comme un nodule ferme, une plaque atrophique ou une lésion sclérodermiforme. Au stade nodulaire, cette lésion initiale s´étale et devient irrégulière, réalisant au bout de quelques mois à quelques années, une masse multinodulaire, souvent polychrome, dure et mobile [2,4]. La transformation en une forme fibrosarcomateuse maligne peut être soit de Novo, soit après une longue histoire évolutive de la maladie [1].

L´échographie montre surtout une masse hypoéchogène richement vascularisée au Doppler. Le scanner montre une masse solitaire, bien définie, isodense, cutanée ou sub-cutanée sans calcifications et l´IRM montre une masse bien définie, isointense avec le muscle en T1, et hypointense en T2. Le bilan d´extension radiologique n´est recommandé que pour les patients dont l´examen clinique fait suspecter des métastases, en cas de DFS récurrent et en cas de transformation sarcomateuse [7].

Le traitement du dermatofibrosarcome est chirurgical, la supériorité et l´efficacité de la chirurgie sans équivoque. La difficulté de la chirurgie réside dans l´extension infra-clinique de la tumeur. La technique la plus répandue est celle de l´exérèse étendue (WLE: Wide local excision) avec une marge de sécurité de 3 à 5cm en latéral et en profondeur avec un sacrifice d´une barrière anatomique saine [1,6,8,9], sans curage ganglionnaire [3,10]. Histologiquement, la tumeur est considérée comme un sarcome de bas grade de malignité faite d´une prolifération cellulaire dense, mal limitée, non encapsulée, occupant le derme le plus souvent dans sa totalité, et envoie de fins prolongements parfois très profonds dans l´hypoderme, ce qui explique les récidives, l´épiderme est respecté et les cellules sont disposées en faisceaux rayonnants (aspect en Rayon de Roue) très évocateurs [1,3,4].

Pour la radiothérapie, le DFS étant à faible activité mitotique, n´est pas radiosensible. Néanmoins elle trouve sa place pour les récidives, les localisations multiples, les tumeurs de grande taille ou primaire inopérable, les localisations empêchant une exérèse large et surtout après une marge d´exérèse insuffisante ou envahie. La chimiothérapie n´est pas une méthode efficace, toutefois un certain protocole avec l´imatinib (Glivec), le pazorpenib ou l´anthracycline peut être utilisé [1,3,5].

Les recommandations du *National Cancer Institute* peuvent guider la prise en charge selon l´évolution de la tumeur [1]. Il n´y a pas de consensus concernant le rythme de surveillance qui doit être axée sur l´examen clinique et maintenu dans le temps du fait de l´évolution lente et du pouvoir récidivant de cette tumeur. Le suivi peut se faire chaque 3 à 6 mois pendant les 5 premiers années puis sera annuel. Parfois on aura recours à une IRM dans quelques cas sélectionnés [8].

Les facteurs prédictifs de récidives les plus évoqués sont: la taille, une poussée évolutive rapide et surtout la qualité d´exérèse initiale [3,4,6].

## Conclusion

Il s´agit d´un cas de jeune patient sans antécédents qui présente une tumeur cutanée rare (DFS) qui se situe entre le pôle de bénignité du fibrome cutané et le pôle de malignité du fibrosarcome cutané vrai. Cette tumeur présente souvent un retard de diagnostic vu son évolution lente et sa méconnaissance par la plupart du cadre médical. L´exérèse chirurgicale d´emblée large et profonde est le traitement de choix. Le pronostic est évalué sur les facteurs de récidive. La surveillance postopératoire régulière et prolongée est nécessaire pour la détection des récidives et la transformation sarcomateuse exceptionnelle.

## Remerciements

Un grand merci au Dr Asma Nasfi anatomopathologiste et Dr Omar Kammoun pour leur aide précieuse.
